# Association between Socio-Demographics and Alcohol Dependence among Individuals Living in an Indian Setting

**DOI:** 10.5539/gjhs.v6n3p16

**Published:** 2014-01-23

**Authors:** B. T. Vignesh, Awnish K. Singh, S. K. Mohan, Shruti Murthy, Ashish Joshi

**Affiliations:** 1 Saveetha Medical College & Hospital, Saveetha University, Chennai, India; 2 Foundation of Healthcare Technologies Society, New Delhi, India; 3 Centre of Global Health and Development, College of Public Health, University of Nebraska Medical Centre, Omaha, USA

**Keywords:** alcohol drinking, alcohol dependence, motivation, socio-demographics, alcohol quitting

## Abstract

**Background:**

Alcohol use is on the rise worldwide and urgent steps are required to curb this growing burden of alcohol consumption. Alcohol drinking leads to serious social, physical and mental consequences.

**Objective:**

The objective of this pilot study is to examine association between socio-demographics and severity of alcohol dependence among individuals obtaining treatment at alcohol de-addiction center.

**Methods:**

This pilot cross sectional study was conducted in September 2013 in South India. A convenient sample of 100 participants was enrolled. Individuals aged 30 years and above, receiving treatment from de-addiction center and providing written informed consent were eligible for the study. A modified version of previously validated questionnaires was used for gathering information on socio-demographic characteristics, severity of alcohol dependence (using Alcohol Dependent Scale [ADS] and Short Alcohol Dependence Data questionnaire [SADD]), motivational incentives for alcohol quitting and challenges faced while quitting alcohol.

**Results:**

All participants were males with mean age of 43 years (SD = 6.5 years). Significant association was seen between ADS and annual income (p = 0.001), education (p = 0.001), occupation (p < 0.0001) and work timing (p < 0.0001). Similar results were seen with SADD scores. Family support (100%) and health (60%) were reported to be the most important motivating factors for quitting alcohol.

**Discussion:**

Results showed an urgent need of interventions that are family centered and focus on unskilled, less educated individuals having high work stress. Public health interventions should not only be home based, but should also include worksite awareness initiatives. A national policy is needed to promote alcohol quitting and to bring awareness regarding the consequences of alcohol consumption on individual’s life.

## 1. Introduction

Alcohol drinking is common practice in many parts of the world. Nevertheless, its consumption leads to serious social, physical and mental consequences (World Health Organization [WHO], 2011). Prior studies have shown that alcohol use exposes the individual to acute health conditions like road traffic accidents and risk of acquiring chronic diseases. Alcoholism is a progressive disease in which individual has been unable to quit drinking and continues to drink even after knowing its harmful effects. Individual faces problem in controlling the drinking, is preoccupied with alcohol, is drinking more to get the desired effect and if stops drinking, individual will face alcohol withdrawal symptoms (Mayo clinic, 2012; Hoffman & Tabakoff, 1996). An alcoholic can’t predict the amount, duration and consequences of alcohol drinking consistently (Mayo clinic, 2012; “Alcoholism”, 2013).

The worldwide prevalence of alcohol use disorders among the individuals attained at the age of 15 years and above was estimated to range from 0% to 16% (WHO, 2004). The highest prevalence was observed in Eastern Europe. It was depicted that the proportion of males with alcohol use disorder was estimated to be highest in Eastern European countries, in parts of South-East Asia and in some countries in the Americas. In Eastern European countries, selected countries in the Americas and Western Pacific Region were estimated of having highest prevalence of alcohol use disorders among females (WHO, 2004). Global per capita consumption is found to be 6.13 liters for individuals aged 15 or above. More than quarter (28.6%) of it is homemade and illegally produced alcohol. It was found that from the total recorded alcohol consumption, spirits are highest (45.7%), followed by beer (36.3%) and wine (8.6%). All other beverages share 10.5% of the total recorded alcohol (“Alcoholism”, 2013).

The American Medical Association classifies alcoholism to have 2 components including both physical and mental. Physiological mechanism that leads to the condition of alcoholism is not well understood (“Alcoholism”, 2013). Socio-demographics, mental health and family history are potential influencers for the risk of alcoholism (Chen, Storr, & Anthony, 2009). It is revealed in research that individuals with moderate drinking habit are less likely to develop an alcohol use disorder. The drinking level for men are less than 4 drinks in a day and less than 14 drinks in a week and for women it is less than 3 drinks and less than 7 drinks in a week (National Institute of Health [NIH]). Individuals drinking above these levels are considered heavy or “at risk” drinkers and if an individual alcohol concentration level reaches 0.08g/dl in blood in 2 hours of drinking, it is considered as binge drinking (NIH).

Several research studies have been done In India to estimate the prevalence of alcohol use (Gururaj, Murthy, Girish & Benegal, 2011). Previous study shows that 35% individuals reported alcohol use in past year and 14% were consuming regularly (Gururaj et al., 2011; John et al., 2009). Similarly results of another study showed that 15% of the respondents consumed alcohol under the influence of familial status or peer pressure (John et al., 2009; Sarangi, Acharya, & Panigrahi, 2008). Findings of another study in the urban slums of Faridabad showed the prevalence of alcohol consumption to be 26% in males in the year 2006 (Gururaj et al., 2011). Countrywide survey of households for Alcohol and drug abuse (2003) showed that the prevalence of alcohol use was 21.4% (Sarkar et al., 2013). An earlier study done on individuals visiting de-addiction center in West Bengal found that 85% of the participants consuming alcohol were in the age group of 20-49 years (Sarkar et al., 2013). A prior study in the past has shown that 25% of the patients with bipolar and alcohol use disorder had attempted suicide (Oquendo et al., 2010). Prior study has shown that college students with parental history of alcohol-related problems drink more or have more alcohol related problems than their peers from non-alcoholic families (Gupta, Saxena, Pednekar, & Maulik, 2003). In another study, age of initiation of alcohol drinking was found to be strong predictor of alcohol misuse at age 17-18 (Baer et al., 2002). Results have also shown that people with alcohol dependence take sick leave more frequently than other employees (Hawkins et al., 1997). In India about 40% of work accidents have been attributed to alcohol use (Skinner & Horn, 1984) and family members may suffer with substantial mental health problems such as depression, irritability and anxiety (Hawkins et al., 1997). Alcohol use disorder leads to unemployment, lower wages and medical expenses along with legal charges which in turn force the individual to live poor social life (Hawkins et al., 1997). The average estimate of social and economic cost of alcohol use in industrialized countries ranges from 1.1% of GDP in Canada to 5-6% in cases of Italy (Hawkins et al., 1997). With increasing purchasing power developing countries like India are not far behind in alcohol misuse. Considering the impact of alcohol abuse in the developing nation like India where alcohol is one of the major sources of revenue there is a special need for understanding the various methods to control its consumption and for promoting individuals to quit it.

To our knowledge, our pilot study is the first of its kind in India to examine association between the socio-demographics and the severity of alcohol dependence among individuals obtaining treatment for alcohol use disorder.

## 2. Methods

A pilot cross sectional study was performed during September 2013 at outpatient department (OPD) of Saveetha Medical College, Chennai, India. A convenient sample of 100 participants was enrolled in this pilot study. Individuals age 30 years and above and receiving treatment from OPD for alcohol use disorder were eligible to participate in the study. Participants were informed about the study purpose before their enrollment in the study. Individuals who were mentally or physically challenged were excluded from participating in the study. All the individuals visiting the OPD for treatment of alcohol use disorder were approached and among them who were satisfying the eligibility criteria and provided the written consent were enrolled in the study until the desired sample of 100 was reached. In the process 28 individuals who were found eligible but refused to participate were excluded from the study. The study protocol was approved by the Institutional Review Board of the Foundation of Healthcare Technologies Society, New Delhi (IRB#FHTS/031/2013) and conforms to the provisions of the Declaration of Helsinki (as revised in Tokyo 2004).

### 2.1 Data Collection Tools

Information on the following variables was gathered in the study by face to face interview of the participants.

(a) **Socio-demographic characteristics**: Information was gathered about age (years), gender, educational status [Grade 1-5, Grade 6-8, Grade 9-10, Grade 11-12, Graduate or above and no education], marital status (single/married/divorce or separated/widow), annual household income, household location or settings(rural, urban), type of family (joint, nuclear, broken, extended), number of family members, occupation status (semi-professional to professional, skilled worker, unskilled, unemployed) (Kumar, Gupta, & Kishore, 2012) and work shift timings (morning, evening, night, alternate, day).
(b) **Alcohol Dependence Scale (ADS):** The ADS provides a quantitative measure of the severity of alcohol dependence consistent with the concept of the alcohol dependence syndrome. The 25 items cover alcohol withdrawal symptoms, impaired control over drinking, awareness of a compulsion to drink, increased tolerance to alcohol, and salience of drink-seeking behavior. The ADS is widely used as a research and clinical tool, and studies have found the instrument to be reliable and valid. It is a 25 item questionnaire used to gather information regarding the alcohol dependence. Dichotomous items were scored 0, 1; three-choice items were scored 0, 1, 2; and four-choice items were scored 0,1,2,3. In each case higher the value greater the dependence. Total score ranges from 0-47 (Skinner & Horn, 1984).
(c) **Short Alcohol Dependence Data Questionnaire (SADD):** It is used to measure the severity of alcohol dependence (Rosa-Oliveria et al., 2011). Behavioral and subjective aspects of alcohol dependence, with adequate construct validity and high correlation with other instruments. The SADD seems to be relatively immune to social-cultural influences and can be used in different settings (Rosa-Oliveria et al., 2011). Information was gathered regarding the alcohol dependence by using 15 items questionnaire (Never, sometimes, often and nearly always). Items were scored from 0-3. The score of 15 items was summed to get the range of 0-45. Scale totals were interpreted as follows: 1-9 low dependence, 10-19 medium dependence, and 20 or greater high dependence (Moderation Management [MM]).
(d) **Incentives and Challenges:** Information was gathered using 2 item open ended questionnaire that recorded the possible motivational reasons for quitting alcohol and the associated challenges faced in quitting alcohol.

### 2.2 Statistical Analysis

Descriptive analysis was performed using univariate statistics to report means and standard deviations for the continuous variables and frequency distribution for the categorical variables. Chi-square analysis was performed to compare the frequency of categorical variables. Correlation and analysis of variance was performed to determine association between alcohol severity and socio-demographic characteristics. Content analysis of the open ended data was performed to identify the common themes that emerged from the analysis. All analysis was performed using SPSS version 16.

## 3. Results

Results show that all of the participants were male with average age of the participants was 43 years (SD = 6.5), most of them were married, having nuclear family with an average family size of 4 (SD = 1). 68% of them were from rural areas. Half of the participants had less than primary education. 58% of the participants were skilled worker, half of the respondents were working in morning shift with an average annual household income of 93,737 INR (SD = 102133) (Table 1).

**Table 1:** Socio-demographics characteristics

Variables	Results
**Socio-Demographics**	
Age(years)	Mean= 43; SD=6.5
**Type of Family**	
Nuclear	90 (90%)
Joint	9 (9%)
Missing	1 (1%)
**Household location or setting**	
Rural	68 (68%)
Urban	30 (30%)
Missing	2 (2%)
**Family size**	Mean=4; SD=1
<5	61 (61%)
≥5	38 (38%)
Missing	1 (1%)
Annual household income, INR*	Mean =93,737; SD=1,02,133
<50000	12 (12%)
50000-60000	41(41%)
>60000	46 (46%)
Missing	1(1%)
**Marital Status**	
Married	90 (90%)
Single	6 (6%)
Divorced/Separated	1(1%)
Missing	1(1%)
**Highest Education level of participant**	
Primary (1^st^-5^th^)	51(51%)
Middle (6^th^-8^th^)	11(11%)
High school (9^th^-10^th^)	15(15%)
Intermediate (11^th^-12^th^) or equivalent	13(13%)
Graduate or Postgraduate	6 (6%)
Missing	4(4%)
**Occupation**	
Skilled worker	58(58%)
Unskilled worker	41(41%)
Missing	1(1%)
**Shift of work**	
Morning	51(51%)
Evening	9(9%)
Night	12(12%)
Alternate	7(7%)
Day	9(9%)
Missing	12(12%)

* INR: Indian National Rupees

Results showed that the average ADS score was 23 (SD = 6). Results showed that 39% of the participants drank enough to get drunk during their last time of drinking. 27% of the participants reported drinking throughout the day. 50% of them reported having hangovers on Sunday or Monday mornings. 76% of the participants use to gulp drinks and 59% of them cannot stop after one drink. 54% of the participants reported frequent trembling of hands and 55% of them had the tendency to have physical sickness as a result of drinking. 45% of the participants agreed to have reported several episodes of delirium tremens. 59% of them reported delusion once or several times after drinking. More than half of them (57%) had fear of not having drink at the time of need. Only 16% of the participants never had blackouts (“loss of memory” without passing out) as a result of drinking and 21% of them had blackouts that lasted a day or more. 66% of them reported tachycardia and palpitation once or several times (Table 2).

**Table 2:** Alcohol dependence scale (ADS)

Alcohol Dependence Scale	Results (%)
*How much did you drink the last time you drank?*	
Enough to get high or less	28 (28%)
Enough to get drunk	39 (39%)
Enough to pass out	32 (32%)
Missing	1 (1%)
*Do you often have hangovers on Sunday or Monday mornings?*	
No	50 (50%)
Yes	50 (50%)
Have you had the “shakes” when sobering up (hands tremble, shake inside)?	
No	22 (22%)
Sometimes	24 (24%)
Often	54 (54%)
Do you get physically sick (e.g., vomit, stomach cramps) as a result of drinking?	
No	18(18%)
Sometimes	55(55%)
Almost every time I drink	27 (27%)
*Have you had the “DTs” (delirium tremens) – that is, seen, felt or heard things not really there; felt very anxious, restless, and over excited?*	
No	55(55%)
Sometimes	34(34%)
Several times	11(11%)
*When you drink, do you stumble about, stagger, and weave?*	
No	23(23%)
Sometimes	42(42%)
Often	34(34%)
Missing	1(1%)
As a result of drinking, have you felt overly hot and sweaty (feverish)	
No	27 (27%)
Once	32(32%)
Several times	41(41%)
*As a result of drinking, have you seen things that were not really there?*	
No	41(41%)
Once	26(26%)
Several times	33(33%)
*Do you panic because you fear you may not have a drink when you need it?*	
No	42(42%)
Yes	57(57%)
Missing	1(1%)
*Have you had blackouts ("loss of memory" without passing out) as a result of drinking?*	
No, never	16(16%)
Sometimes	39(39%)
Often	32(32%)
Almost every time I drink	13(13%)
*Do you carry a bottle with you or keep one close at hand?*	
No	39(39%)
Some of the time	39(39%)
Most of the time	22(22%)
*After a period of abstinence (not drinking), do you end up drinking heavily again?*	
No	29(29%)
Sometimes	47(47%)
Almost every time I drink	24(24%)
*In the past 12 months, have you passed out as a result of drinking?*	
No	31(31%)
Once	40(40%)
More than once	27(27%)
Missing	2(2%)
*Have you had a convulsion (fit) following a period of drinking?*	
No	32(32%)
Yes	37(37%)
Several times	31(31%)
*Do you drink throughout the day?*	
No	73(73%)
Yes	27(27%)
*After drinking heavily, has your thinking been fuzzy or unclear?*	
No	14(14%)
Yes, but only for a few hours	35(35%)
Yes, for one or two days	32 (32%)
Yes, for many days	19 (19%)
*As a result of drinking, have you felt your heart beating rapidly?*	
No	34 (34%)
Yes	46 (46%)
Several times	20 (20%)
*Do you almost constantly think about drinking and alcohol?*	
No	40 (40%)
Yes	59 (59%)
Missing	1(1%)
*As a result of drinking, have you heard “things” that were not really there?*	
No	32 (32%)
Yes	39 (39%)
Several times	29 (29%)
*Have you had weird and frightening sensations when drinking?*	
No	26 (26%)
Once or twice	46 (46%)
Often	28 (28%)
*As a result of drinking have you “felt things” crawling on you that were not really there (e.g., bugs, spiders)?*	
No	55(55%)
Yes	30 (30%)
Several times	15(15%)
*With respect to blackouts (loss; of memory)*	
Have never had a blackout	19 (19%)
Have had blackouts that last less than an hour	40 (40%)
Have had blackouts that last for several hours	20 (20%)
Have had blackouts that last a day or more	21(21%)
*Have you tried to cut down on your drinking failed?*	
No	39 (39%)
Once	43(43%)
Several times	18(18%)
*Do you gulp drinks (drink quickly?)*	
No	24(24%)
Yes	76(76%)
After taking one or two drinks, can you usually stop?	
No	59 (59%)
Yes	41(41%)

The average SADD scores was 18 (SD = 4). Results showed that 61% of the participants felt that sometimes getting drunk is more important than the next meal while 33% of them agreed that they drink always in morning, afternoon and evening. More than half of them (53%) reported that sometimes they drink as much as they want irrespective of their next day assignments. 42% of the participants drink alcohol irrespective of its affects. Thirty-four per cent of the participants knew that they won’t be able to stop drinking after beginning while 39% of them never tried to control it. 78% of them reported forgetting things in next morning after having heavy drinks (Table 3).

**Table 3:** Short Alcohol Dependence Data (SADD) questionnaire

SADD	Results (%)				
	Never	Sometime	Often	Nearly always	Missing
Do you rind difficulty in getting the thought of drinking out of your mind?	17(17%)	73(73%)	8(8%)	2(2%)	
Is getting drunk more important than your next meal?	14(14%)	61(61%)	18(18%)	6(6%)	1(1%)
Do you plan your day around when and where you can drink?	26(26%)	33(33%)	35(35%)	6(6%)	
Do you drink in the morning, afternoon and evening?	26(26%)	19(19%)	21(21%)	33(33%)	1(1%)
Do you drink for the effect of alcohol without caring what the drink is?	39(39%)	26(26%)	19(19%)	15(15%)	1(1%)
Do you drink as much as you want irrespective of what you are doing the next day?	22(22%)	53(53%)	14(14%)	11(11%)	
Given that many problems might be caused by alcohol do you still drink too much?	18(18%)	26(26%)	42(42%)	13(13%)	1(1%)
Do you know that you won’t be able to stop drinking once you start?	20(20%)	27(27%)	17(17%)	34(34%)	2(2%)
Do you try to control your drinking by giving it up completely for days or weeks at a time?	39(39%)	28(28%)	21(21%)	12(12%)	
The morning after a heavy drinking session do you need your first drink to get yourself going?	29(29%)	49(49%)	10(10%)	12(12%)	
The morning after a heavy drinking session do you wake up with a definite shakiness of your hands?	25(25%)	24(24%)	44(44%)	7(7%)	
After a heavy drinking session do you wake up and retch or vomit?	38(38%)	24(24%)	13(13%)	24(24%)	1(1%)
The morning after a heavy drinking session do you go out of your way to avoid people?	35(35%)	28(28%)	26(26%)	10(10%)	1(1%)
After a heavy drinking session do you see frightening things that later you realize were imaginary?	22(22%)	49(49%)	20(20%)	8(8%)	1(1%)
Do you go drinking and the next day rind you have forgotten what happened the night before?	22(22%)	28(28%)	30(30%)	20(20%)	

### 3.1 Stratified Analysis

The ADS scores were stratified to 4 categories to assess the level of alcohol dependence It was found that majority (65%) of the individuals had substantial level of alcohol dependence (score 22-30), 15% had intermediate level of alcohol dependence (score 14-21), 13% had low level of alcohol dependence (score 1-13) and 3% had severe level of alcohol dependence (score 31-47). Similar results were seen with the SADD scores. It was found that 46% of the study participants had high dependence (score of ≥ 20), 40% had medium dependence (score 10-19), and 6% had low dependence (score 1-9).

### 3.2 Association between Socio-Demographics and ADS and SADD Scores

Results showed significant association between ADS and annual income (p = 0.001), education (p < 0.0001) and work timing (p < 0.0001). Similar results were seen for SADD scores (Table 4). In addition, marital status (p = 0.04) and occupation (p = .008) was also found significantly associated with SADD scores. No other significant association was seen with ADS or SADD scores.

**Table 4:** Association between Socio-demographics and ADS and SADD scores

Socio-demographic characteristics	ADS* Score	SADD^‡^Score
Age	r=0.14; p=0.14	r=0.14; p=0.17
Family size	r=0.01; p=0.85	r=0.13; p=0.19
Annual income	r=-0.34; p=0.001	r=-0.29; p=0.005
Family type	F=2.3; p=0.10	F=1.2; p=0.3
Location	F=2.1; p=0.12	F=0.8; p=0.43
Marital status	F=2.1; p=0.09	F=2.8; p=0.04
Education	F=5.6; p<0.0001	F=8.7; p<0.0001
Occupation	F=2.8; p=0.07	F=5; p=0.008
Work timings	F=5; p<0.0001	F=4.2; p=0.002

* ADS: Alcohol Dependence Scale

‡ Short Alcohol Dependence Data questionnaire (r= Pearson correlation coefficient; F= F test statistic of ANOVA)

Results of various ADS categories showed that individuals with less education (80%, n = 41; p< .0001) and work timings of evening (100%, n = 9; p = .01) or alternate shifts (92%, n = 11; p = .01) were significantly associated with high alcohol dependence. Family type (p = .23), location (p = .13), marital status (p = .12) and occupation (p = .07) were not significantly associated with high alcohol dependence.

**Figure 1:**
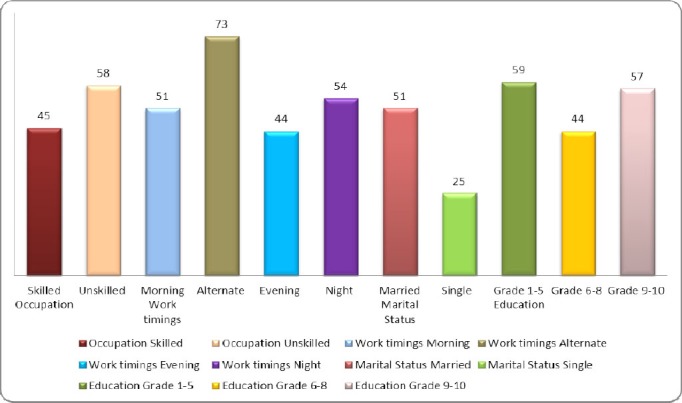
Frequency distribution of high alcohol dependence individuals

Similar results were seen among individuals with high alcohol dependence using various score categories of SADD. Participants who were married (51%, n = 43; p = .008), less educated (59%, n = 29; p< .0001) and working in night (54%, n = 7; p = .04); alternate (73%, n = 8; p = .04) or evening (44%, n = 4; p = .04) shifts (Figure 1), showed significant alcohol dependence. Occupation (p = .08), Family type (p = .10) and household location (p = .40) did not show any significant association.

### 3.3 Motivations for Leaving Alcohol

Results showed that 100% of the individuals reported that family support was the biggest motivation for them to quit alcohol use. Health (60%) was the second most important motivating factor for individuals to quit alcohol. Results showed that family centered intervention with increased awareness about the health consequences after high consumption of alcohol are needed. Other motivations included access to better surroundings or environment (27%), or by increasing the costs of alcohol purchases (16%). The major challenge for the individuals not able to quit alcohol was due to increased work stress (52%), addiction to alcohol (46%), peer pressure (14%) and emotional behaviors (14%). The results suggest need for an intervention programs that should not only be home based but should also include work site initiatives that will help individuals to reduce alcohol dependence.

## 4. Discussion

Alcohol use is on the rise worldwide and urgent steps are required to curb this growing burden of alcohol consumption (Smith, 2008). Our pilot study is an attempt to evaluate the severity of alcohol dependence among the alcoholics visiting the OPD of Saveetha Medical College in Chennai, India. To our knowledge, this is the first study to evaluate the association between socio-demographics and alcohol dependence scores in an Indian population. Results showed that more than half of the population had either medium level of alcohol dependence (40%) or high level alcohol dependence on SADD (46%). More than half of them (65%) had substantial level of alcohol dependence and 3% of them had severe level of alcohol dependence on ADS. This shows very high severity of dependence on alcohol among the participants. Almost all individuals visiting the OPD for treatment of alcohol use disorder were male which can be attributed to social barriers, obstructing the females to visit OPD fro treatment.

Past study in Mumbai, reported 19% current use of alcohol among the participants and history of past use was reported in 5% of the participants (WHO, 2004).

Results of our study showed significant association between SADD Score and annual income (p = 0.005), marital status (p = 0.04), education (p < 0.0001), occupation (p = 0.008) and work timings (p = .002). Similar results were seen with ADS and showed significant associations between ADS Score and annual income (p = 0.001), education (p = 0.001) and work timings (p < 0.0001). Globally it was observed that people with alcohol dependence take sick leave more frequently than other employee’s (WHO, 2004). In past study it was found that men with lower education and lower standard of living were more likely to report a risky usual quantity of alcohol (C60g/drinking day) (Pillai et al., 2012). Past study showed that 55% caregivers of alcohol dependent patients experienced it as a mild to moderate burden and 45% felt it as a significant burden (Swapna, Sudarshan, & Begum, 2012). In our study, family support, increased health awareness and greater financial burden were identified as the most common factors that would possibly motivate the individuals to quit alcohol. Stress and addiction were two major challenges faced by the participants in quitting alcohol. A study in slums of Kolkata revealed that 84% of the individuals were not concerned about their level of consumption (Ghosh, Samanta, & Mukherji, 2012). Our study showed that 12% of the individuals tried constantly to control drinking by giving it up completely for days or weeks at a time.

There were several limitations associated with our study. First it included smaller sample size, and the study design was cross sectional. This limits establishing causality and can only describe associations between the various variables and alcohol dependence. Further the study was limited to one geographical location so the results of the study cannot be generalized. Also the study included only males so the findings of our study may not be applicable to females. Study didn’t included confounding factors like family history of alcohol consumption and childhood environment and their consideration in future studies will give deeper insight of the issue.

Results showed an urgent need of interventions that are family centered, target individuals who are unskilled, less educated, and have high work stress. Public health interventions should not only be home based but should also include awareness initiatives at the worksite. There is an urgent need to conduct a longitudinal study to examine a temporal relationship between high alcohol dependence and various multiple factors including socio-demographics, environmental, behavioral and work site related variables. A national policy needs to be formulated aimed to create awareness about moderation of alcohol consumption, associated health consequences, impact on family and friends and work productivity.
